# Structure and Function of Oral Microbial Community in Periodontitis Based on Integrated Data

**DOI:** 10.3389/fcimb.2021.663756

**Published:** 2021-06-17

**Authors:** Zhengwen Cai, Shulan Lin, Shoushan Hu, Lei Zhao

**Affiliations:** ^1^ State Key Laboratory of Oral Diseases, West China College of Stomatology, Sichuan University, Chengdu, China; ^2^ National Clinical Research Center for Oral Diseases, West China College of Stomatology, Sichuan University, Chengdu, China; ^3^ Department of Periodontics, West China Hospital of Stomatology, Sichuan University, Chengdu, China

**Keywords:** 16S, periodontitis, bacteria, microbiome, metabolite, biomarker, high-throughput nucleotide sequencing

## Abstract

**Objective:**

Microorganisms play a key role in the initiation and progression of periodontal disease. Research studies have focused on seeking specific microorganisms for diagnosing and monitoring the outcome of periodontitis treatment. Large samples may help to discover novel potential biomarkers and capture the common characteristics among different periodontitis patients. This study examines how to screen and merge high-quality periodontitis-related sequence datasets from several similar projects to analyze and mine the potential information comprehensively.

**Methods:**

In all, 943 subgingival samples from nine publications were included based on predetermined screening criteria. A uniform pipeline (QIIME2) was applied to clean the raw sequence datasets and merge them together. Microbial structure, biomarkers, and correlation network were explored between periodontitis and healthy individuals. The microbiota patterns at different periodontal pocket depths were described. Additionally, potential microbial functions and metabolic pathways were predicted using PICRUSt to assess the differences between health and periodontitis.

**Results:**

The subgingival microbial communities and functions in subjects with periodontitis were significantly different from those in healthy subjects. *Treponema, TG5*, *Desulfobulbus*, *Catonella*, *Bacteroides*, *Aggregatibacter*, *Peptostreptococcus*, and *Eikenella* were periodontitis biomarkers, while *Veillonella*, *Corynebacterium*, *Neisseria*, *Rothia*, *Paludibacter*, *Capnocytophaga*, and *Kingella* were signature of healthy periodontium. With the variation of pocket depth from shallow to deep pocket, the proportion of Spirochaetes, Bacteroidetes, TM7, and Fusobacteria increased, whereas that of Proteobacteria and Actinobacteria decreased. Synergistic relationships were observed among different pathobionts and negative relationships were noted between periodontal pathobionts and healthy microbiota.

**Conclusion:**

This study shows significant differences in the oral microbial community and potential metabolic pathways between the periodontitis and healthy groups. Our integrated analysis provides potential biomarkers and directions for in-depth research. Moreover, a new method for integrating similar sequence data is shown here that can be applied to other microbial-related areas.

## Introduction

Periodontitis is an inflammatory condition affecting periodontal tissue and is the result of uncontrolled gingivitis (Chapple et al., 2018). The pathogenesis of periodontitis remains unclear, and the diagnosis of periodontitis heavily depends on the clinical manifestation and periodontal detection indicators (periodontal probing depth, clinical attachment loss, bleeding on probing, and alveolar bone loss) (Kinane et al., 2017; Papapanou et al., 2018). At present, it is widely acknowledged that dental plaque is the key initiating factor. Destruction of the periodontium is attributed to microbial dysbiosis as well as the excessive immune response of the hosts (Kinane et al., 2017). The transition from healthy gingiva to periodontitis occurs because of the accumulation of pathogenic microorganisms and is affected by multiple risk factors such as modifiable habits and immutable genetic predisposition, eventually leading to oral ecological disturbance (Genco and Borgnakke, 2013; Papapanou et al., 2018). However, no specific pathobionts have yet been identified. This may be because we have overlooked specific pathobionts that are low in number, or that an imbalanced microbiota is the underlying cause, rather than specific pathobionts. For instance, the red complex in subgingival plaque (*Porphyromonas gingival*, *Treponema denticola*, and *Tannerella forsythia*) play an important role in periodontal dysbiosis (Socransky et al., 1998).

Previous studies have analyzed the correlation between microorganisms and periodontitis. Nevertheless, *in vitro* bacterial cultures and polymerase chain reaction (PCR)-based analysis alone limit the ability to observe the complex profiles of subgingival microbiota. Next-generation sequencing technology has been widely used in microecology, which has the ability to present the whole appearance of microbiota and enable researchers to explore multiple dimensions of microbial communities (Gu et al., 2019). Combined with conserved 16S ribosomal RNA targeted assays, the external interference by host genes can be eliminated, and it can accurately identify and quantify various microorganisms (Cummings et al., 2016). The evolutionary and taxonomic relationships among microorganisms can also be evaluated, in addition to predicting their characteristic properties and metabolic pathways (Langille et al., 2013).

Several studies have been conducted on human oral microbiota in subjects with healthy periodontal tissue, gingivitis, and different states of periodontitis. Samples are collected from different sites including the supragingival area (Galimanas et al., 2014), subgingival plaque (Tsai et al., 2018), saliva (Chen et al., 2018), and gingival crevicular fluid (Pei et al., 2020). The current trend is to explore target samples to identify sensitive pathogenic biomarkers by microbiology, metabolomics, or multiomics (Su et al., 2020). However, studies in a small sample can be disturbed by diversified confounders that may lead to biased conclusions. Therefore, there should be a way to use existing data to realize the hidden information mining, aimed at identifying the common microbial characteristics of periodontitis. To this end, we screened high-quality sequence datasets, merged, and processed data under a unified standard. Finally, we analyzed oral microbial communities to identify the common characteristic microorganisms as well as the functions and potential metabolic pathways in different periodontitis patients.

## Materials and Methods

### Microbiome Data Source Collection and Eligibility Criteria

All included microbial datasets were retrieved according to the predetermined design. Relevant studies and data were collected by December 30, 2020, through electronic databases: (1) The National Library of Medicine (MEDLINE by PubMed) was searched using the following keywords: ((((periodontitis) OR (chronic periodontitis)) OR (aggressive periodontitis)) OR (periodontal disease)) AND (16S) AND (subgingival). (2) The Genomes of National Center for Biotechnology Information was searched using the following search strategy: 16S [All Fields] AND periodontitis [All Fields] AND subgingival [All Fields]. The search results yielded 919 publications. All abstracts were browsed and selected by two authors (Z.W. Cai and S.L. Lin) to remove duplicate and non-clinical original articles. Only subgingival microbial sequencing-related studies were retained. The full text of 38 studies was obtained. We searched for the details including study design information, inclusion criteria of participants, and the results of clinical periodontal indicators, to assess whether the inclusion criteria and the results of clinical periodontal indices were consistent with our predetermined criteria, which were based on the latest classification of periodontal disease (Chapple et al., 2018; Papapanou et al., 2018). The inclusion criteria for the periodontitis group were as follows: (1) periodontal probing depth (PPD) >4 mm, (2) clinical attachment loss (CAL) >3 mm, and (3) bleeding on probing (BOP) at >10% of sites. The inclusion criteria for healthy individuals were as follows: (1) without PPD or PPD <4 mm, (2) mean CAL <2 mm, and (3) mean BOP <10%. The exclusion criteria for all subjects were (1) pregnancy or systemic disease, (2) periodontal therapy sought within the past 3 months, and (3) use of antibiotics in the past 3 months. Twenty-two studies were eliminated because of unavailable sequence datasets or because they did not meet our predetermined criteria (**Supplementary Material 1**). Sixteen articles were included and their datasets downloaded from NCBI or ENA.

### Raw Sequence Processing and Re-Filter

**Figure 1:**
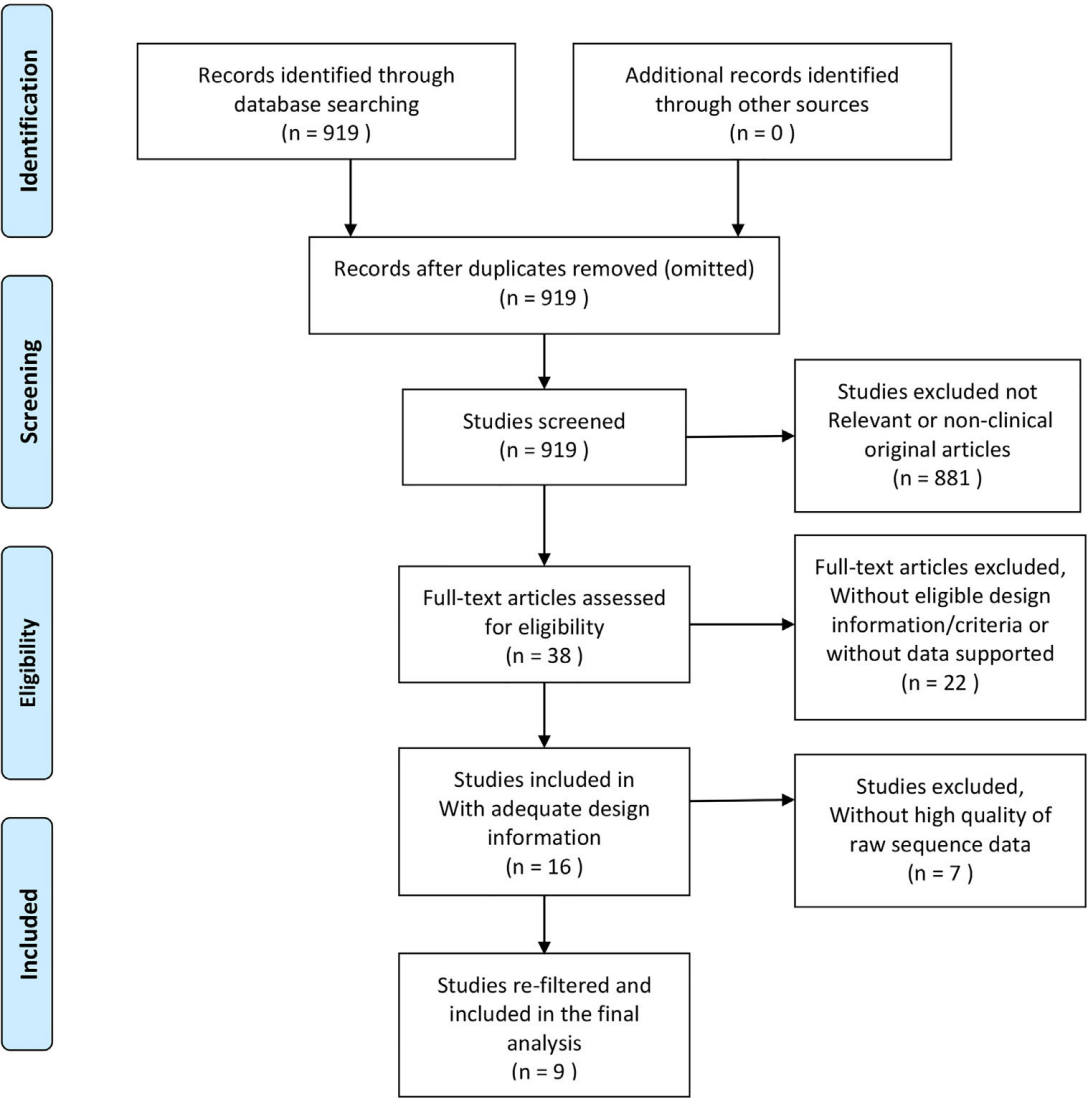
Modified flow diagram of collecting and screening articles, processing, and re-filtering data.

The 16 raw sequence datasets were merged and processed with a uniform standard *via* QIIME2 pipeline version 2020.8 using default parameters (Bolyen et al., 2019). DADA2 (Wolf and Evans, 2018) was used to denoise the data and assess the sequence quality score (QS). The parameters for trimming and truncation settings were 10 and 150, respectively. Samples with overall quality <25 were eliminated. Then, the amplicon sequence variants (ASVs, obtained from DADA2) sequence data were clustered into operational taxonomic units (OTUs) at 97% similarity in closed-reference of the Greengenes database gg-13-8 version (DeSantis et al., 2006), and the Feature Table was created. Through this step, we matched the different short reads with the representative sequence. The short reads without a matching representative sequence in the library were eliminated, and the representative sequence taxa were classified with a trained Naïve Bayesian classifier for their annotations (Wang et al., 2007). Next, we re-filtered the Feature Table to eliminate samples with microbial features <4 or the frequency of microorganism <1000. Finally, seven more publications were eliminated because they were not suited for further analysis (**Supplementary Material 1**); nine datasets were included in the final analysis (**Table 1**). The risk of bias in the included studies was assessed according to the Downs-Black checklist (**Supplementary Material 2**) (Downs and Black, 1998). Two authors (Z.W. Cai and S.S. Hu) independently assessed the risk of bias, and any disagreement was resolved through consultation with the third author (L. Zhao). The quality level of eight studies was fair, and one study was of good quality. The average score of the nine articles was 16.25, and the equality level was fair. **Figure 1** shows the flow diagram of the process of literature selection.

**Table 1:** Summary of the studies included in pooled analysis.

Author	Accession	Sample-source	Region	Description (Number of participants)	
				HC	PD
Califf et al.	PRJEB19122	Sub, Supra	V4V5	–	34
Galimanas et al.	PRJEB6047	Sub, Supra	V3	11	13
Bizzarro et al.	PRJNA289294	Sub	V5-V7	–	37
Griffen et al.	SRP009299	Sub	V1V2/V4	–	29
Wei et al.	PRJNA509532	Sub, Buccal mucosa	V4V5	9	23
Shi et al.	SRP228020	Sub, GCF	V4	10	24
Liu et al.	SRP102224	Sub	V3V4	–	12
Pérez et al.	PRJNA324274	Sub	V3	7	9
Chen et al.	SRP075100	Sub, Saliva	V4	21	48

PD, periodontal disease; HC, healthy control; Sub, subgingival plaque; Supra, supragingival plaque; GCF, gingival crevicular fluid.

Some samples in those sequence datasets were removed due to not meeting the inclusion criteria.

### Datasets for Analysis

The datasets from nine publications (Griffen et al., 2012; Galimanas et al., 2014; Bizzarro et al., 2016; Califf et al., 2017; Chen et al., 2018; Pérez-Chaparro et al., 2018; Wei et al., 2019; Liu et al., 2020; Shi et al., 2020) were divided into two groups—periodontal disease (PD) and healthy control (HC)—according to our predetermined criteria. The mean age of the HC group was 34.4 ± 7.1 years, and the mean age of the PD group was 43.7 ± 8.2 years. Some samples collected from different sites (such as supragingival dental plaque, tongue, and saliva) were excluded. Only subgingival samples were included for the analysis, which could better demonstrate the relationship between microorganisms and periodontitis. Other irrelevant confounding factors were also eliminated as much as possible: data of patients with periodontitis who were post-treated with sodium hypochlorite in Califf’s study were deleted. The patients treated with antibiotics in Bizzarro’s study or post-treated with no-surgical periodontal treatment in Chen’s study were removed. The healthy group in Griffen’s study was excluded because some participants’ BOP was >10%. Moreover, three studies (Bizzarro et al., 2016; Califf et al., 2017; Pérez-Chaparro et al., 2018) with definite parameters of periodontal pocket depth were selected to explore the relationship between clinical periodontal pocket depth and the variation of microbiota. The other six studies were not included because there were no specific pocket depth parameters supported in the original metadata. The subgingival samples were divided into the following four groups based on the periodontal pocket depth: 0–3 mm (healthy control); 3–4 mm; 5–6 mm; and 7–9 mm.

### Statistics Analysis

The microbial structure was evaluated by alpha diversity (Shannon diversity index, Observed features vector, Simpson diversity index, and Chao1 index) and beta diversity (Jaccard distance, Bray-Curtis distance, unweighted UniFrac distance, and weighted UniFrac distance matrix) based on a rarifying sample depth of 1000 in QIIME2 (Bolyen et al., 2019). Beta diversity was tested by multivariate homogeneity of dispersions (PERMDISP) (Anderson, 2006) and permutational multivariate analysis of variance (PERMANOVA) (Anderson, 2001) to test the homogenous dispersion and variance between groups. Microbial composition analysis was carried out on the Microbiome-Analyst platform (Chong et al., 2020). Default parameters were used to preprocess the data, which included a count filter and a variance filter. The count filter removed the samples in small number, and the variance filter deleted the constant features in each group. Centered log ratio (CLR) transformation was performed for normalization prior to data analysis. The Mann–Whitney U test was selected as the default statistical test. Linear discriminant analysis effect size (LEfSe) was adopted for the microbial comparison analysis (significance level, p<0.05 and linear discriminant analysis [LDA] score >2 were chosen to characterize the phenotype) (Segata et al., 2011). Correlation network analysis used Spearman’s rank correlation with the threshold set to 0.3. Microbial community function was predicted *via* PICRUSt (Langille et al., 2013) to explore the potential interactions among host, environment, and microbial community. The principle was to match the whole genome of the corresponding homologous ancestor through 16S sequencing, and then map it to metabolites as well as pathways to achieve functional prediction. The enrichment pathway analysis of the PD and HC groups was on level 2 and 3 functional dimensions based on the Kyoto Encyclopedia of Genes and Genomes (KEGG) database (www.kegg.jp). RNA-seq methods (Algorithm: edgeR, adjusted p-value cut-off: <0.05) were adopted to analyze the significant difference in microbial functions and microbiota at different pocket depths (Robinson et al., 2010).

## Results

### Oral Microbial Structure and Composition

**Figure 2:**
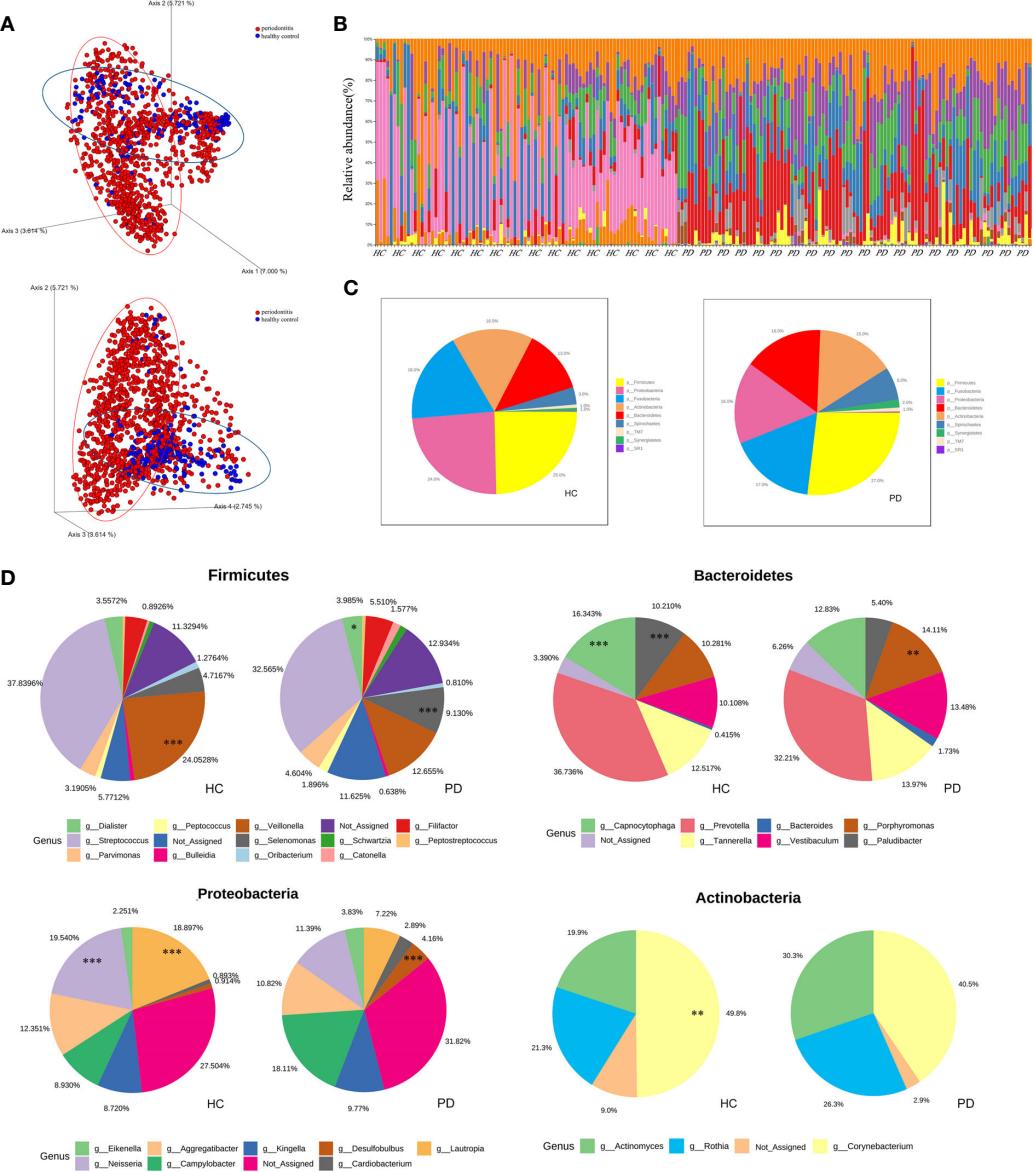
**(A)** The 3D-PCoA plot based on Jaccard distance matrix (p<0.001, PERMANOVA) illustrates the beta diversity of oral microbiota. PD (periodontitis, red spots), HC (healthy control, blue spots). **(B)** The taxonomic bar plots on the phylum level. Each column represents a sample (HC left and PD right), and each small fragment in different colors represents different phyla. **(C)** The pie charts demonstrate the difference of microbial composition between HC (left) and PD (right) on the phylum level. Different colors correspond to the phyla on the list. **(D)** The subcategories composition of four phyla. Each pair of pie charts shows the comparison of microbial abundance between HC and PD on the genus level (*p < 0.05; **p < 0.01; ***p < 0.001, tested by RNA-seq methods, algorithm: edgeR).

**Figure 3:**
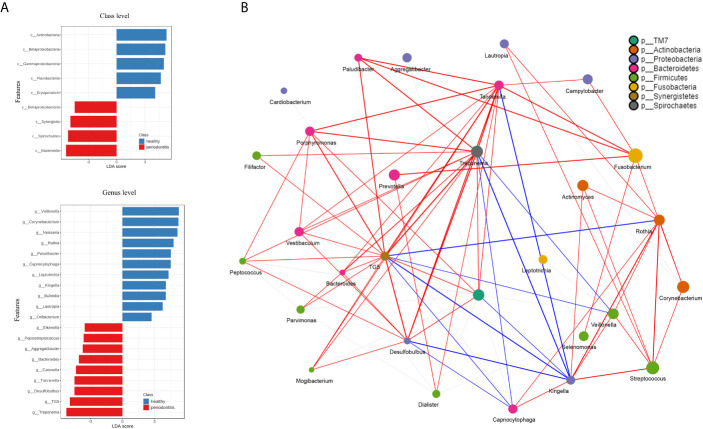
**(A)** The ordinate is the taxa with significant differences between the groups, and the abscissa is a bar graph to visually display the LDA analysis logarithmic score value of each taxa. The longer the length, the more significant the difference in the taxon. Red bars indicate periodontitis and blue bars indicate healthy control. **(B)** The network analysis shows the correlation between microorganisms on the genus level. Each genus is colored according to its phylum. The edges show greater correlations and the node size reflects the abundance. Red and blue lines represent positive and negative correlations, respectively.

The beta diversity (Jaccard distance matrix, p<0.001, PERMANOVA) demonstrated the clusters of subgingival microbial structure between the PD and HC groups (**Figure 2A**). It also showed inhomogeneous dispersion between both groups (Jaccard distance matrix, p<0.001, PERMDISP), which means that both location effect and dispersion effect existed. Other beta diversity analyses are presented in **Supplementary Figure 1A**. The alpha diversity Chao1 and observed index showed significant difference (p<0.01), whereas the Simpson and Shannon index showed no difference between HC and PD (**Supplementary Figure 1B**). A taxonomic bar plot was used to compare the profile of HC and PD groups at the phylum level (**Figure 2B**). It was observed that the microbial community in PD was more complex than that in HC, and the proportions of several phyla changed, which was regarded as a state of dysbiosis. The pie chart shows differences in microbial composition between HC and PD on the phylum level (**Figure 2C**). The dominant phyla of PD were Firmicutes (27%), Fusobacteria (17%), Proteobacteria (16%), Bacteroidetes (16%), Actinobacteria (15%), and Spirochaetes (6%). The dominant phyla of HC were Firmicutes (25%), Proteobacteria (24%), Fusobacteria (18%), Actinobacteria (16%), Bacteroidetes (13%), and Spirochaetes (3%). To illustrate the subtle differences in the composition of HC and PD, we compared the composition of subcategories of some phyla between HC and PD separately: Firmicutes, Proteobacteria, Bacteroidetes, and Actinobacteria (**Figure 2D**). In Firmicutes, the genus *Veillonella* (p<0.001) increased in HC, whereas *Selenomonas* and *Dialister* (p<0.001) were seen more abundantly in PD. In Proteobacteria, the genus *Neisseria* (p<0.001) and *Lautropia* (p<0.001) were found abundantly in HC, while *Desulfobulbus* (p<0.001) was rich in PD. In Bacteroidetes, the proportion of *Porphyromonas* (p<0.01) increased, whereas *Capnocytophaga* (p<0.001) and *Paludibacter* (p<0.001) decreased in PD. In phylum Actinobacteria, *Corynebacterium* (p<0.01) increased in HC. LEfSe was used to identify significant differences in taxa. (1) On the class level: the biomarkers of PD were Bacteroidia, Spirochaetes, Synergistia, and Deltaproteobacteria, while Actinobacteria, Betaproteobacteria, and Flavobacteria were the biomarkers of HC. (2) On the genus level: *Treponema, TG5*, *Desulfobulbus*, *Catonella*, *Bacteroides*, *Aggregatibacter*, *Peptostreptococcus*, and *Eikenella* were biomarkers for periodontitis, while *Veillonella*, *Corynebacterium*, *Neisseria*, *Rothia*, *Paludibacter*, *Capnocytophaga*, and *Kingella* were biomarkers for the healthy group (**Figure 3A**).

### Microbial Correlation Network

Correlation network analysis among the microbial community is shown in **Table 2**. The characteristic genera of PD, i.e., *Treponema*, *Tannerella, TG5*, *Desulfobulbus*, *Porphyromonas*, *Treponema*, and *Filifactor* were positively correlated with each other (**Figure 3B**). There were also positive correlations between normal oral bacteria and healthy-related microbes (*Kingella*, *Capnocytophaga*, *Rothia*, *Veillonella*, *Streptococcus*, and *Corynebacterium*). Negative correlations were between oral normal microbes and periodontal pathobionts. For example, *TG5*, *Treponema*, *Tannerella*, and *Desulfobulbus* were negatively correlated with *Kingella* as well as *Veillonella*. More relationships among the microbial community are presented in **Supplementary Material 3**.

**Table 2:** Correlation network analysis of the microbial community.

Taxon1	Taxon2	Correlation	P.value	Statistic
*Treponema*	*TG5*	0.7191	<0.01	32561635.83
*Desulfobulbus*	*TG5*	0.6455	<0.01	41094316.01
*Tannerella*	*Treponema*	0.5774	<0.01	48986356.52
*Desulfobulbus*	*Treponema*	0.5446	<0.01	52793791.74
*Tannerella*	*TG5*	0.5364	<0.01	53736459.74
*Capnocytophaga*	*Kingella*	0.5071	<0.01	57139373.92
*Kingella*	*Rothia*	0.4634	<0.01	62197661.63
*Corynebacterium*	*Rothia*	0.4582	<0.01	62806659.08
*Streptococcus*	*Veillonella*	0.4462	<0.01	64198665.84
*Rothia*	*Streptococcus*	0.4448	<0.01	64356577.13
*Dialister*	*Prevotella*	0.4427	<0.01	64602407.19
*Desulfobulbus*	*Tannerella*	0.4417	<0.01	64720393.22
*Corynebacterium*	*Lautropia*	0.4357	<0.01	65409187.11
*Filifactor*	*Treponema*	0.4325	<0.01	65785509.61
*Peptococcus*	*Treponema*	0.429	<0.01	66187131.79
*Actinomyces*	*Rothia*	0.4137	<0.01	67957682.15
*Porphyromonas*	*Tannerella*	0.4076	<0.01	68666527.88
*Paludibacter*	*Treponema*	0.4049	<0.01	68985946.96
*Campylobacter*	*Fusobacterium*	0.4016	<0.01	69367430.26
*Desulfobulbus*	*Vestibaculum*	0.3982	<0.01	69759006.29
*Mogibacterium*	*TG5*	0.3982	<0.01	69763542.92
*Porphyromonas*	*TG5*	0.393	<0.01	70363518.18
*Paludibacter*	*Tannerella*	0.3885	<0.01	70882356.24
*Mogibacterium*	*Treponema*	0.3855	<0.01	71235324.49
*Fusobacterium*	*Selenomonas*	0.3812	<0.01	71724014.35
*Bacteroides*	*TG5*	0.3793	<0.01	71948445.92
*Porphyromonas*	*Treponema*	0.377	<0.01	72212475.12
*Desulfobulbus*	*Mogibacterium*	0.3711	<0.01	72897777.71
*Rothia*	*TG5*	-0.3042	<0.01	151183647.29
*Capnocytophaga*	*Desulfobulbus*	-0.3091	<0.01	151744548.94
*Desulfobulbus*	*Kingella*	-0.325	<0.01	153592374.07
*TG5*	*Veillonella*	-0.3299	<0.01	154162465.49
*Kingella*	*Tannerella*	-0.3476	<0.01	156211678.52
*Treponema*	*Veillonella*	-0.3685	<0.01	158629770.93
*Capnocytophaga*	*Treponema*	-0.375	<0.01	159392440.50
*Capnocytophaga*	*TG5*	-0.3865	<0.01	160722624.16
*Kingella*	*TG5*	-0.4239	<0.01	165050919.50
*Kingella*	*Treponema*	-0.4479	<0.01	167842107.45

Correlation network analysis on genus level used Spearman rank correlation with the threshold set at 0.3. Only part of the correlation is presented.

### Microbial Composition Changing With PPD

**Figure 4:**
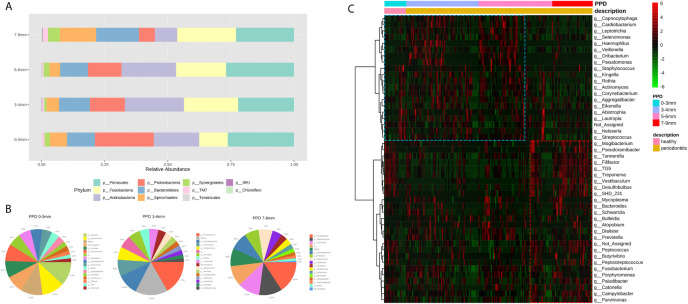
**(A)** The taxonomic bar plots on the genus level. Each bar represents a group of different periodontal probing depth (0–3 mm, 3–4 mm, 5–6 mm, and 7–9 mm). **(B)** Three pie charts display the genera (abundance >1%) in the sites of PPD 0–3 mm, 3–4 mm, and 7–9 mm. The taxonomy “others” is a cluster of genera whose abundance are less than 1%. **(C)** The heatmap shows the correlations between taxa and PPD. Each column represents a sample, each row represents a taxon, and each lattice represents a correlation coefficient between a taxon and PPD group (red lattice, positive correlation; green lattice, negative correlation). With the deepening of pocket depth, the pathobionts increase (red box) and some normal microbes decrease (blue box).

The taxa plot displayed the microbial abundance of different pocket depths on the phylum level. With the variation of PPD from shallow to deep pockets, the proportion of Spirochaetes, Bacteroidetes, TM7, and Fusobacteria increased, whereas Proteobacteria and Actinobacteria decreased (**Figure 4A**). The microbial composition of HCs (PPD: 0–3 mm), shallow layer (PPD: 3–4 mm) group, and deep layer (PPD: 7–9 mm) group are shown on the genus level in **Figure 4B**. Healthy controls consisted of *Neisseria*, *Streptococcus*, and some other bacteria. The shallow layer group consisted of *Fusobacterium, Corynebacterium*, *Actinomyces*, *Streptococcus*, and some other bacteria. In the deep layer (PPD: 7–9 mm) group, *Fusobacterium*, *Porphyromonas*, and *Treponema* were the dominant genera. The comparison of microorganisms in the deep layer and the shallow layer are presented in **Table 3**. In the deep layer, *Desulfobulbus*, *TG5*, *SHD_231, Tannerella*, *Porphyromonas*, and some other pathobionts increased significantly, whereas *Pseudomonas*, *Haemophilus*, *Actinomyces*, *Capnocytophaga*, and some oral normal bacteria decreased significantly. A heatmap was used to show the correlations among different taxa and PPD (**Figure 4C**). The correlation coefficient between the pathobionts (such as *Mogibacterium*, *Tannerella*, *Filifactor*, *TG5*, *Treponema*, *Desulfobulbus*, and *Peptostreptococcus*) and the deep periodontal pockets is higher than the correlation coefficient between these pathobionts and the shallow pocket depth. These pathobionts illustrated an increasing trend with the deepening of pocket depth. In contrast, some microorganisms such as *Corynebacterium*, *Rothia*, *Kingella*, *Neisseria*, and *Haemophilus* had a higher correlation coefficient in the groups with healthy (PPD: 0–3 mm) and shallow (PPD: 3–4 mm) pocket depths. These normal microbes displayed a decreasing trend with the pocket depth.

**Table 3:** Comparison of microorganisms in the deep layer (PPD: 7–9 mm) and the shallow layer (PPD: 3–4 mm).

Taxon	log2FC	LogCPM		P values	FDR
*Desulfobulbus*	3.7079	13.03	<0.001		<0.001
*TG5*	3.2558	14.706	<0.001		<0.001
*Pseudomonas*	-7.4192	16.079	<0.001		<0.001
*Haemophilus*	-6.0582	13.966	<0.001		<0.001
*Eubacterium*	2.983	10.262	<0.001		<0.001
*SHD_231*	3.1276	10.859	<0.001		<0.001
*Filifactor*	3.3358	14.779	<0.001		<0.001
*Tannerella*	2.5596	15.028	<0.001		<0.001
*Treponema*	2.4324	16.494	<0.001		<0.001
*Lautropia*	-4.2751	15.045	<0.001		<0.001
*Actinomyces*	-2.9909	16.941	<0.001		<0.001
*Capnocytophaga*	-3.0637	13.944	<0.001		<0.001
*Streptococcus*	-2.3346	16.191	<0.001		<0.001
*Mogibacterium*	2.1055	10.219	<0.001		<0.001
*Abiotrophia*	-3.1646	10.654	<0.001		<0.001
*Corynebacterium*	-3.0281	17.348	<0.001		<0.001
*Peptococcus*	2.045	11.502	<0.001		<0.001
*Rothia*	-3.3356	16.289	<0.001		<0.001
*Vestibaculum*	2.9434	14.144	<0.001		<0.001
*Neisseria*	-3.4407	14.256	<0.001		<0.001
*Leptotrichia*	-2.344	14.919	<0.001		<0.001
*Kingella*	-2.703	13.456	<0.001		<0.001
*Peptostreptococcus*	2.0265	10.73	<0.001		<0.001
*Veillonella*	-2.2552	14.784	<0.001		<0.001
*Oribacterium*	-2.1402	10.499	<0.001		<0.001
*Aggregatibacter*	-2.5911	13.703	<0.001		<0.001
*Porphyromonas*	1.5748	15.452	<0.001		<0.001
*Mycoplasma*	1.4871	10.702	<0.01		<0.01
*Bacteroides*	1.4567	11.735	<0.01		<0.01
*Cardiobacterium*	-1.702	12.594	<0.01		<0.01
*Parvimonas*	1.2651	13.251	<0.01		<0.01
*Selenomonas*	-0.94956	15.082	<0.05		<0.05
*Fusobacterium*	0.54974	17.479	0.086998		0.11185
*Butyrivibrio*	0.71479	9.2838	0.098256		0.12282
*Dialister*	-0.76662	13.014	0.11462		0.1394
*Paludibacter*	0.59143	13.375	0.16633		0.19697
*Bulleidia*	-0.58357	10.01	0.21877		0.25243
*Campylobacter*	0.29517	14.966	0.38402		0.43202
*Eikenella*	-0.26099	12.195	0.56464		0.61973
*Schwartzia*	0.20297	12.061	0.61726		0.64448
*Staphylococcus*	-0.10987	9.9382	0.80758		0.80758

Log2FC (log2 fold change) represents the ratio of two groups (PPD 7–9 vs. PPD 3–4) based log2. LogCPM (log counts per million) represents the expression level of variables. FDR is the false discovery rate as correction of P value.

### Microbial Community Functions Analysis

The KEGG functional orthologs (KOs) were gathered to comprehensively analyze the involved enrichment pathways. The function and pathways of microbes between the PD and HC groups were compared on different functional dimensions. At level 2, cell motility, cellular processing and signaling, nucleotide metabolism, metabolism of cofactors and vitamins, and nervous system function were significantly different (p<0.05). At level 3, bacterial motility proteins and flagellar assembly increased in the PD group. There were some differences in amino acid metabolism (e.g., tyrosine, histidine, D-arginine, D-ornithine, and glycine) between the HC and PD groups. Synthesis and degradation of ketone bodies, nitrogen metabolism, and sulfur metabolism were also significantly different (p<0.05). Part of the significant functions and pathways are shown in **Table 4**. More details about the differential analysis on levels 2 and 3 are provided in **Supplementary Material 4** and **5**. The random forest model distinguished PD from HC almost without error (class error<0.01), implying special functions and metabolic pathways of periodontitis-related microbiota (**Supplementary Figure 2**).

**Table 4:** Significant functions and pathways on L2 and L3 compared with periodontitis and healthy groups.

Variables (L2)	log2FC	LogCPM	P values	FDR
Cell Motility	0.3819	14.092	<0.001	<0.001
Environmental Adaptation	0.10176	10.496	<0.001	<0.01
Signal Transduction	0.087304	13.795	<0.001	<0.01
Metabolism of Terpenoids and Polyketides	-0.02687	14.146	<0.001	<0.01
Metabolism of Cofactors and Vitamins	-0.02879	15.525	<0.001	<0.01
Folding, Sorting and Degradation	-0.02063	14.726	<0.001	<0.01
Cellular Processes and Signaling	-0.0408	15.158	<0.01	<0.05
Nervous System	-0.07875	9.5907	<0.01	<0.05
Variables (L2)	log2FC	LogCPM	P values	FDR
Bacterial motility proteins	0.40809	13.024	<0.001	<0.001
Bacterial chemotaxis	0.48527	11.742	<0.001	<0.001
Methane metabolism	0.088374	13.439	<0.001	<0.001
Flagellar assembly	0.58686	11.586	<0.001	<0.001
Ether lipid metabolism	0.56359	5.9541	<0.001	<0.001
Other ion-coupled transporters	-0.060619	13.597	<0.001	<0.001
Xylene degradation	0.28929	8.4607	<0.001	<0.01
Pentose and glucuronate interconversions	0.11254	11.654	<0.001	<0.01
Carotenoid biosynthesis	-0.43936	6.6335	<0.001	<0.01
Ubiquitin system	-0.40326	7.6626	<0.001	<0.01
Nitrogen metabolism	-0.040959	12.795	<0.001	<0.01
Insulin signaling pathway	0.082719	9.7838	<0.001	<0.01
D-Arginine and D-ornithine metabolism	-0.29459	5.8248	<0.001	<0.01
Base excision repair	-0.046719	12.258	<0.001	<0.01
Sulfur metabolism	-0.12471	11.39	<0.01	<0.05
Polycyclic aromatic hydrocarbon degradation	-0.077289	10.401	<0.01	<0.05
Tyrosine metabolism	-0.056456	11.869	<0.01	<0.05
Synthesis and degradation of ketone bodies	0.20517	9.0478	<0.01	<0.05
Histidine metabolism	0.053466	12.395	<0.01	<0.05
Linoleic acid metabolism	0.16046	8.5719	<0.01	<0.05
Chloroalkane and chloroalkene degradation	0.090688	10.423	<0.01	<0.05
Carbon fixation pathways in prokaryotes	0.034009	13.338	<0.01	<0.05
Primary immunodeficiency	-0.070153	9.19	<0.01	<0.05
Glycine, serine, and threonine metabolism	0.0204	13.083	<0.01	<0.05
Type I diabetes mellitus	-0.05003	9.2339	<0.01	<0.05

Log2FC (log2 fold change) represents the ratio of two groups (PD vs. HC) based log2.

LogCPM (log counts per million) represents the expression level of variables.

FDR is the false discovery rate as correction of P value.

## Discussion

### Overall Review

Our study elucidated the subgingival microbial structure of periodontitis patients *via* integrated datasets. Extensive literature searches and rigorous screening criteria were performed. Datasets were merged and processed using uniform standards for eventual analysis. Subgingival microbial community, periodontitis biomarkers, potential functions of microbiota, and their collaborative network were also evaluated. Furthermore, we described the variation of microbial composition in different PPDs. Our results showed that some pathobionts were consistent with those reported in previous studies (Kirst et al., 2015; Liu et al., 2020) and supported the finding that periodontal dysbiosis was not due to specific microorganisms, rather due to the increasing level of pathobionts. The two reasons for this are likely that (1) microbial community dysbiosis leads to periodontal disease, and (2) periodontitis is caused by some specific pathogenic bacteria that have not yet been identified.

### Microorganisms Associated With Periodontitis

Compared with previous studies and the included subgroup studies, our analysis yielded some consistent results and novel potential periodontitis-related microbes. *Porphyromonas*, *Treponema*, and *Tannerella* were found closely related to periodontitis. Moreover, with the deepening of PPD, *Porphyromonas* and *Treponema* occupied the main components of subgingival microbes, while the healthy periodontium-related genera *Neisseria* and *Lautropia* decreased. The abundance of *Spirochaetes*, *Synergistes, Desulfobulbus*, and *Bacteroides* also increased in PD. These results were consistent with those reported by (Galimanas et al., 2014; Califf et al., 2017; Pérez-Chaparro et al., 2018). Healthy gingiva-associated genera *Rothia*, *Capnocytophaga*, *Veillonella*, *Corynebacterium*, and *Neisseria* were found in our results, which were also partly reported by (Galimanas et al., 2014; Bizzarro et al., 2016; Chen et al., 2018). Notably, Proteobacteria appeared to be a point of contention with different reports in several articles. In Shi’s and Griffen’s studies, Proteobacteria was higher in healthy controls than in periodontitis patients. By contrast, Galimanas reported that Proteobacteria was associated with periodontitis, although he later noted that Proteobacteria was associated with the healthy population in subgingival microbiota. Our findings showed that Proteobacteria was more closely related to HC and the proportion of Proteobacteria decreased with the deepening of the periodontal pocket. Additionally, we identified some potential genera associated with periodontitis, such as *TG5* and *Catonella*, whose relationship with periodontitis has been rarely reported; thus, more trials are required to validate their pathogenic mechanisms in periodontitis.

LEfSe analysis showed that *Corynebacterium* and *Rothia* were biomarkers for healthy periodontium. *Rothia* is among the normal genera that colonize the oral cavity. Although it was detected in some opportunistic infectious diseases (Ramanan et al., 2014), our findings support its classification as a typical oral bacterium. This is consistent with Meuric’s research (Meuric et al., 2017) that the ratio of *Porphyromonas*, *Treponema*, and *Tannerella* to *Rothia* and *Corynebacterium* is an excellent predictor of periodontitis, which regards *Corynebacterium* and *Rothia* as non-pathogenic genera. *Veillonella*, *Kingella*, and *Neisseria* were thought to be healthy biomarkers. *Veillonella* can consume the lactic acid produced by *Streptococcus mutans* to prevent caries (Sanz et al., 2017). In a clinical trial of periodontal therapy, *Kingella* and *Veillonella* were found to be more associated with therapeutic success (Colombo et al., 2012). For *Neisseria*, its abundance declined from HCs and the shallow layer to the deep layer in periodontal pocket, which showed it was a biomarker for healthy periodontium.

Our results showed that *Desulfobulbus*, *Treponema*, and *Tannerella* were periodontitis biomarkers. The correlation between *Desulfobulbus* and periodontitis was discovered recently, represented by *Desulfobulbus oralis*, which has not been valued previously owing to limitations in culture and isolation (Cross et al., 2018). It was found that *D. oralis* can directly induce the inflammatory response in oral epithelial cells to promote the occurrence of periodontitis. *Treponema denticola*, *Porphyromonas gingivalis*, and *Tannerella forsythia* belong to the red complex, which are considered to be the most periodontitis-related microbial aggregation (Socransky et al., 1998). In our results, *Porphyromonas* and *Treponema* both displayed significant dominance in the PPD 7–9 mm group with a 11% abundance ratio. Inversely, they were <2% in the PPD 0–3 mm group. Our network correlation analysis also reflected the synergy among these microbes. *P. gingivalis* is critically related to periodontitis. This black anaerobic bacterium relies on its fimbriae, lipopolysaccharides, proteases, and other virulence factors to colonize on teeth and periodontal tissues, and it can co-aggregate a variety of other potential pathogenic microorganisms (Mysak et al., 2014). Additionally, it can interfere with host immune functions such as cytokine secretion, degrade recruitment, and weaken leukocyte defenses in periodontal tissues (Kobayashi-Sakamoto et al., 2003). Several years ago, the subgingival concentration of *Treponema* growth was considered significantly related to PPD and attachment loss (Armitage et al., 1982). *Tannerella* is another pathogenic genus implicated in periodontitis, which is associated with subgingival bleeding and regarded as a risk marker of PD (Suda et al., 2004). The abundance of Fusobacteria increased with increasing PPD. On the genus level, the proportion of *Fusobacterium* changed from 8% in the 0–3 mm PPD group to 16% in the 7–9 mm PPD group. The role of Fusobacteria in deep periodontal pockets cannot be ignored. Its pathogenic ability is to co-aggregate and help periodontal pathobionts to colonize, which acts as a bridge for dental plaque biofilm formation (Rickard et al., 2003). Moreover, *F. nucleatum* can invade epithelial cells to escape host immunity and trigger inflammatory responses, and FadA was recognized as the crucial virulence factor (Han et al., 2000).

*TG5* and *Catonella* were identified as potential periodontitis-related pathobionts in our study. Few studies have reported its existence in patients with periodontitis. Only few research studies have confirmed the virulence and pathogenic mechanisms of *TG5* in periodontitis. *Catonella* is an oral pathobiont associated with oral infections and oral cancer (Zhao et al., 2017). However, its role in the development of periodontal disease remains to be investigated.

In network analysis, we can clearly observe positive correlations among pathobionts, and positive correlations among normal and healthy oral microorganisms. Our results showed that the microbes associated with PD or HC can be classified into two communities. The microorganisms cooperate with others in the same community, but are negatively correlated with the bacteria in the other community. This association was consistent with the results reported by Liu (Liu et al., 2020) and supported the antagonistic relationships between pathogenic and non-pathogenic bacteria. It was worth emphasizing that *Mogibacterium*, *Parvimonas*, and *Filifactor* were all positively correlated with some known pathogenic microorganisms in the correlation analysis, although they were not found in the LEfSe analysis. This may indicate a new direction in the discovery of pathobionts, although their abundance is not high.

### Remarkable Microbial Metabolic Pathways

The differences in metabolic pathways and functions caused by alteration of microbiota were obvious. The increasing levels of bacterial motility proteins and flagellar assembly may imply that the invasion ability of pathobionts plays an important role in periodontitis. The metabolism of tyrosine was significantly different in our results (p<0.01). Liebsch et al. (Liebsch et al., 2019) showed that dental plaque and pocket depth were positively correlated with the metabolites derived from phenylalanine and tyrosine catabolism. For example, phenylacetate, a bacterial metabolite, was significantly associated with periodontal disease and may be a candidate marker for periodontal disease screening (Liebsch et al., 2019). The metabolism level of ketone bodies increased in PD (p<0.05). This was consistent with another study that reported decreased pyruvate and pyruvic acid levels in the saliva of patients with chronic periodontitis (Romano et al., 2018). Sulfur metabolism was different between the PD and HC groups, which may be associated with the production of volatile sulfur compounds (VSCs). These VSCs are known to be produced by anaerobic microbes and are toxic to periodontal tissue (Hampelska et al., 2020). It is also the major reason for halitosis in patients with periodontitis. A longitudinal study evaluated the correlation between periodontitis progress and VSCs, and the results showed a positive association between the two (Makino et al., 2012).

### Limitations and Prospect

In this study, we determined the biomarkers of periodontitis based on the abundance of microorganisms; however, our results are inadequate and more extensive research, such as on virulence factors, is needed to confirm their pathogenesis in periodontitis. At the literature inclusion stage, we did not retrieve all the studies related to subgingival microbiota of periodontitis patients. However, it should be pointed out that the data acquisition and analysis in our study are different from a systematic review and meta-analysis. The biggest hurdle was to access complete and high-quality datasets. Because many datasets are unavailable, it is challenging to obtain all datasets to analyze. The short reads were matched with the Greengenes database library (gg-13-8 version) in Closed Reference way to annotate. However, it screened some unidentified microorganisms in this method. A more complete microbial gene database and powerful computers are needed to improve this analysis. Heterogeneity across studies is a confounding factor. For instance, 16SrRNA sequencing analysis can be biased by PCR. This is inevitable at present, and we can only reduce this heterogeneity through unified criteria and data processing methods. We screened literature with predetermined criteria and only included high-quality datasets for merging, aiming to decrease the influence of multiple variables between different researches as much as possible. Some variables remain inevitably among the similar studies, but the variables from different studies can be minimized, which has been described by (Kirst et al., 2015). Some other studies confirmed that the idea of merging datasets under strict screening criteria and unified sequence data processing can be feasible (Sze and Schloss, 2016; Meuric et al., 2017). Overall, this method could magnify the pathogenic features of periodontitis and minimize variables from different studies, such as individual differences, experimental differences, and technical differences, helping to identify the common pathobionts among different periodontitis patients. Perhaps, a more unified protocol for high-throughput sequencing studies can be designed, which will be conducive to the realization of data aggregation. Additionally, the results of functional profiles predicted from 16S amplicons were not as accurate as those of whole-genome sequencing, which should be further validated (Jing et al., 2021).

## Conclusion

We applied strict and unified standards to process sequence datasets, and analyzed the microbial community structure and functions in periodontitis. The results showed significant differences in the structure of microorganisms and potential functions and metabolic pathways between the PD and HC groups. Furthermore, we revealed that the composition of the subgingival microbiota changed at different PPD sites. Our results identified some potential periodontitis biomarkers and explored the functions of subgingival microbiota in periodontitis. Besides, we described a feasible method to pool microbial sequence data, which can be used in other related areas. With the updates to microbial database and the improvement of sequencing technology, the advantages of this method may be greater, which can be used to identify more unknown and unannotated pathobionts in the future.

## Data Availability Statement

Publicly available datasets were analyzed in this study. This data can be found here: National Center for Biotechnology (NCBI) and The European Nucleotide Archive (ENA): PRJEB19122, PRJEB6047, PRJNA289294, SRP009299, PRJNA509532, SRP228020, SRP102224, PRJNA324274, SRP075100.

## Author Contributions

ZC and LZ contributed to conception and design. ZC and SL contributed to data acquisition, screening, processing, and manuscript drafting. ZC, SL, and SH contributed to analysis and interpretation of data. ZC, SL, SH, and LZ contributed to manuscript revisions. All authors contributed to the article and approved the submitted version.

## Funding

This study is supported by the National Natural Science Foundation of China (Grant No. 81970944 and No.81991502) and a research grant from West China Hospital of Stomatology (LCYJ2019-4).

## Conflict of Interest

The authors declare that the research was conducted in the absence of any commercial or financial relationships that could be construed as a potential conflict of interest.
