# Supplementary material for: Structure and Function of Oral Microbial Community in Periodontitis Based on Integrated Data
Source: Front Cell Infect Microbiol. 2021 Jun 17;11:663756. doi: 10.3389/fcimb.2021.663756 (PMC8248787; doi:10.3389/fcimb.2021.663756)
Supplement: Supplementary file 1 [file DataSheet_1.docx]

Supplementary Material

# Supplementary data

# Supplementary table 1

Supplementary table 1 displays a list of the literature/datasets excluded in the pre-analysis.

# Supplementary table 2

The risk of bias in included studies was assessed according to the Downs–Black checklist. Considering the workability, item 27 rating the study powers was replaced with whether the study did or did not perform power calculation (YES=1, NO=0). Based on the total grades, every included literature was given a corresponding quality level: excellent (26–28), good (20–25), fair (15–19), and poor (≤14).

# Supplementary data 3

Supplementary data 3 displays the correlation network analysis within the microbial community (Spearman rank correlation with the threshold set at 0.3).

# Supplementary data 4 and 5

The differential abundance analysis results between PD and HC on levels 2 and 3 are provided in supplementary data 4 and 5.

L2 shows the differential abundance analysis result on level 2 in Supplementary data 4.

L3 shows the differential abundance analysis result on level 3 in Supplementary data 5.

# Supplementary data 6

The scripts for QIIME2 to merge and process datasets.

# Supplementary Figure

**Supplementary Figure 1**


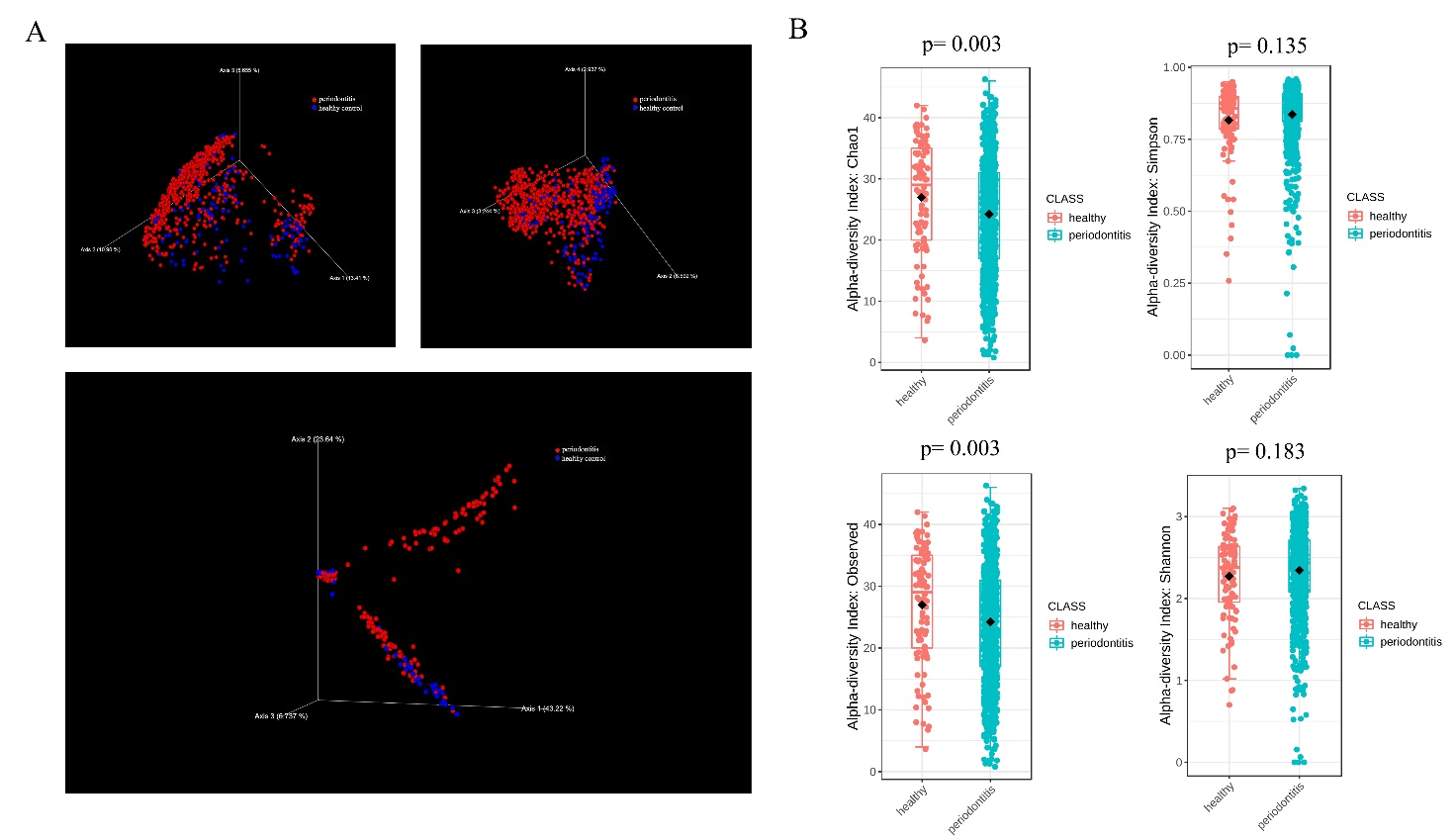


(A) The 3D-PCoA plot illustrates the oral microbiota beta diversity (Bray-Curtis distance, unweighted UniFrac distance, and weighted UniFrac distance matrix). PD (periodontitis, red spots), HC (healthy control, blue spots).

(B) Alpha-diversity index Chao1 and Observed displayed the significant difference between healthy and periodontitis groups on the phylum and genus levels (p<0.01). The Simpson and Shannon index showed no difference between the HC and PD groups.

**Supplementary Figure 2**


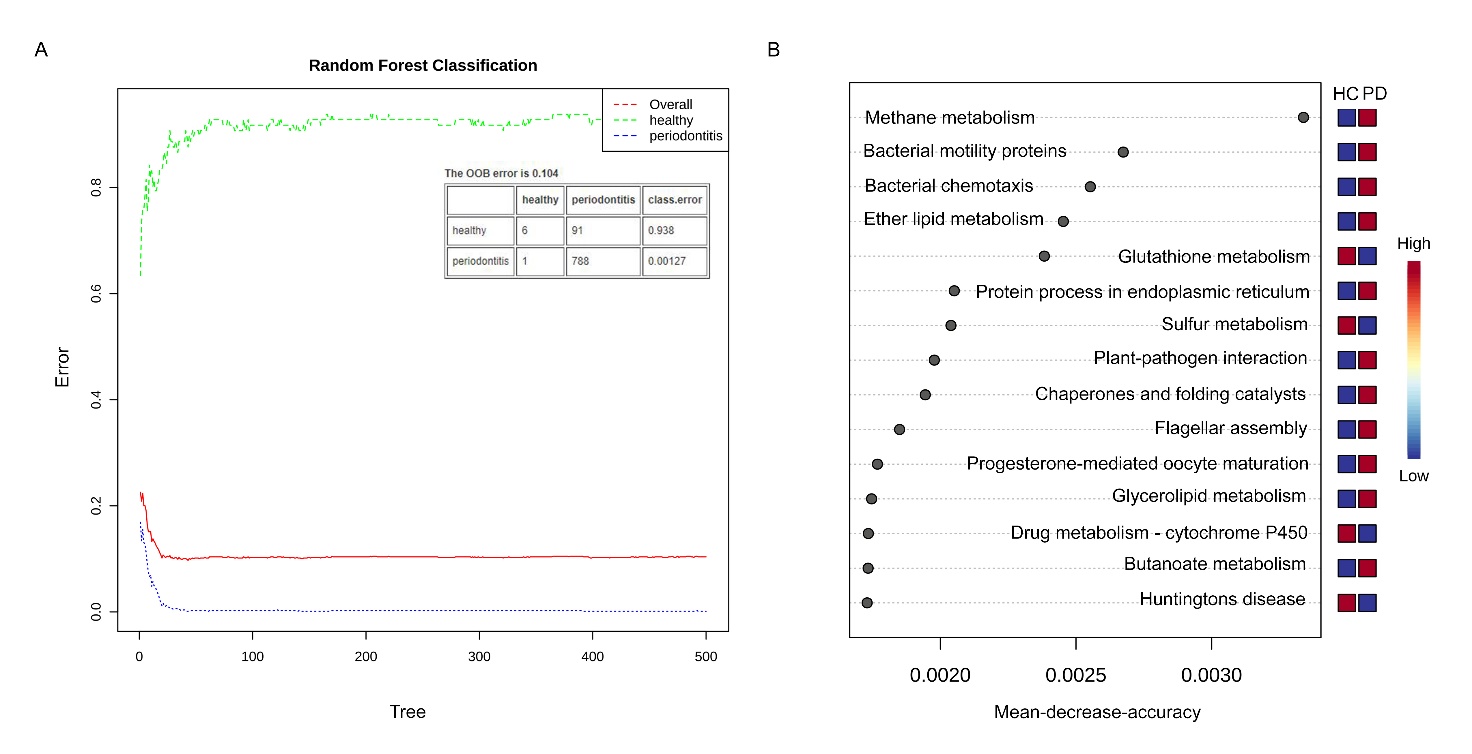


(A) The Random Forest model classified periodontitis accurately (blue line, error <0.01).

(B) Comparison of significantly different functions of microbial community in the periodontitis and healthy groups. The ordinate represents different important functional variables in PD and HC. Red represents higher enrichment of the functional variables and blue represents lower level of variables in the corresponding group. The x-coordinate number is the mean-decrease-accuracy, which is a metrics of variables in classification and shows a large impact on the accuracy of classification. The larger the number, the more important the variable is in judgment of Random Forest Classification.
