# Supplementary material for: Structure and Function of Oral Microbial Community in Periodontitis Based on Integrated Data
Source: Front Cell Infect Microbiol. 2021 Jun 17;11:663756. doi: 10.3389/fcimb.2021.663756 (PMC8248787; doi:10.3389/fcimb.2021.663756)
Supplement: Supplementary file 2 [file Table_1.docx]

Supplementary Table 1

The literature/datasets excluded in the pre-analysis.

Exclude publications (22 of 38)

| Author/name | Title/theme | Accession/link | Reason for exclusion |
| --- | --- | --- | --- |
| Coretti et al. | Subgingival dysbiosis in smoker and non‑smoker patients with chronic periodontitis | PRJEB17957 | Without dataset supported in Accession number |
| A bio-project | A study on the subgingival plaque bacteria community in type 2 diabetic patients. | PRJNA182759 | Out of inclusion criteria |
| Tsai et al. | Subgingival microbiota in individuals with severe chronic periodontitis | PRJNA274944 | Without eligible study design |
| Lu et al. | Microbiome in maintained periodontitis and its shift over a single maintenance interval of 3 months | - | No dataset supported |
| Teng et al. | Cetylpyridinium chloride mouth rinses alleviate experimental gingivitis by inhibiting dental plaque maturation | SRA063171 | No dataset in accession number |
| Valenza et al. | Microbial changes in periodontitis successfully treated by mechanical plaque removal and systemic amoxicillin and metronidazole | - | Out of inclusion criteria |
| Sakamoto et al. | Changes in oral microbial profiles after periodontal treatment as determined by molecular analysis of 16S rRNA genes | - | No dataset supported |
| Acharya et al. | Species-Level Salivary Microbial Indicators of Well-Resolved Periodontitis: A Preliminary Investigation | - | No dataset supported |
| Feres et al. | Microbiome changes in young periodontitis patients treated with adjunctive metronidazole and amoxicillin | - | No dataset supported |
| Kim et al. | Grading system for periodontitis by analyzing levels of periodontal pathogens in saliva | - | Out of inclusion criteria |
| Ge et al. | Oral Microbiome of deep and shallow dental pockets In chronic periodontitis | PRJNA182936 | No dataset in accession number |
| Belstrøm et al. | Influence of periodontal treatment on subgingival and salivary microbiotas | - | No dataset supported |
| Damgaard et al. | Porphyromonas gingivalis in saliva associates with chronic and aggressive periodontitis | - | Out of inclusion criteria |
| Schulz et al. | Comparison of the oral microbiome of patients with generalized aggressive periodontitis and periodontitis-free subjects | - | No dataset supported |
| Lundmark et al. | Identification of Salivary Microbiota and Its Association With Host Inflammatory Mediators in Periodontitis | PRJEB21767 | Out of inclusion criteria |
| Hagenfeld et al. | No differences in microbiome changes between anti-adhesive and antibacterial ingredients in toothpastes during periodontal therapy | PRJEB28345 | Out of inclusion criteria |
| Hagenfeld et al. | Do we treat our patients or rather periodontal microbes with adjunctive antibiotics in periodontal therapy? A 16S rDNA microbial community analysis | PRJEB18651 | Without study design metadata  supported |
| A bio-project | A study on the oral microbiota in periodontitis patients and periodontally healthy people | PRJNA528558 | Without study design metadata  supported |
| Arjunan et al. | Oral Pathobiont Activates Anti-Apoptotic Pathway, Promoting both Immune Suppression and Oncogenic Cell Proliferation | PRJNA279076 | Out of inclusion criteria |
| Hagenfeld et al. | Significant Short-Term Shifts in the Microbiomes of Smokers With Periodontitis After Periodontal Therapy With Amoxicillin & Metronidazole as Revealed by 16S rDNA Amplicon Next Generation Sequencing | PRJEB35812 | Out of inclusion criteria |
| Moon et al. | Subgingival microbiome in smokers and non‐smokers in Korean chronic periodontitispatients | - | No dataset supported |
| Genco et al. | The Subgingival Microbiome Relationship to Periodontal Disease in Older Women | - | No dataset supported |

Exclude publications (7 of 16)

| Author/name | Title/theme | Accession/link | Reason for exclusion |
| --- | --- | --- | --- |
| Pei et al. | Microbial and metabolomic analysis of gingival crevicular fluid in general chronic periodontitis patients: lessons for a predictive, preventive, and personalized medical approach | PRJNA579148 | Small numbers (<1000) of features after DADA2 filtering. Samples are obtained from gingival crevicular fluid. |
| Kirst et al. | Dysbiosis and alterations in predicted functions of the subgingival microbiome in chronic periodontitis | [PRJNA269205](http://www.ncbi.nlm.nih.gov/bioproject/?term=PRJNA269205) | Small numbers (<1000) of features after filtering. |
| A bio-project | A study on Interdental and subgingival microbiota affecting the tongue microbial ecology and oral malodour in health, gingivitis and periodontitis | PRJNA649803 | Low quality of dataset with incomplete metadata. |
| Jünemann et al. | Bacterial community shift in treated periodontitis patients revealed by ion torrent 16S rRNA gene amplicon sequencing | PRJEB3032 | Low quality of dataset |
| Boutin et al. | Clustering of Subgingival Microbiota Reveals Microbial Disease Ecotypes Associated with Clinical Stages of Periodontitis in a Cross-Sectional Study | <https://figshare.com/s/8da5157b93740ae58b8e> | analysis failure when importing the sequence datasets into QIIME2 |
| Schwarzberg et al. | The personal human oral microbiome obscures the effects of treatment on periodontal disease | <http://www.microbio.me/qiime>. ID2083 | Parse failure when demultiplexing the sequence |
| Abusleme et al. | The subgingival microbiome in health and periodontitis and its relationship with community biomass and inflammation | SRA051864 | The dataset is incomplete |
