# Supplementary material for: Structure and Function of Oral Microbial Community in Periodontitis Based on Integrated Data
Source: Front Cell Infect Microbiol. 2021 Jun 17;11:663756. doi: 10.3389/fcimb.2021.663756 (PMC8248787; doi:10.3389/fcimb.2021.663756)
Supplement: Supplementary file 3 [file Table_2.docx]

**Supplement Table 2.** Risk of bias assessment of the studies included by Downs and Black checklist

| Author | TOTAL  (28) | Reporting  (11) | External validity  (3) | Internal validity  (bias) (7) | Selection bias (6) | Power (1) | Equality level |
| --- | --- | --- | --- | --- | --- | --- | --- |
| Califf | 20 | 9 | 2 | 5 | 4 | 0 | good |
| Galimanas | 16 | 9 | 1 | 4 | 2 | 0 | fair |
| Bizzarro | 18 | 9 | 2 | 4 | 3 | 0 | fair |
| Griffen | 16 | 9 | 1 | 4 | 2 | 0 | fair |
| Wei | 15 | 8 | 1 | 4 | 2 | 0 | fair |
| Shi | 17 | 9 | 2 | 4 | 2 | 0 | fair |
| Liu | 15 | 8 | 1 | 4 | 2 | 0 | fair |
| Pérez | 15 | 8 | 1 | 4 | 2 | 0 | fair |
| Chen | 18 | 9 | 2 | 4 | 3 | 0 | fair |
| Average | 16.25 | 8.67 | 1.44 | 4.11 | 2.44 | 0 | Fair |

Quality level: excellent (26-28), good (20-25), fair (15-19) and poor (≤14)
