# Supplementary material for: Structure and Function of Oral Microbial Community in Periodontitis Based on Integrated Data
Source: Front Cell Infect Microbiol. 2021 Jun 17;11:663756. doi: 10.3389/fcimb.2021.663756 (PMC8248787; doi:10.3389/fcimb.2021.663756)
Supplement: Supplementary file 7 [file Table_6.docx]

#datasets import

time qiime tools import \

--type 'SampleData[SequencesWithQuality]' \

--input-path manifest.txt \

--output-path demux.qza \

--input-format SingleEndFastqManifestPhred33V2

time qiime tools import \

--type 'SampleData[PairedEndSequencesWithQuality]' \

--input-format PairedEndFastqManifestPhred33V2 \

--input-path manifest-2.txt \

--output-path demux.qza \

#DADA2 denoise

qiime dada2 denoise-single \

--i-demultiplexed-seqs demux.qza \

--p-trim-left 10 \

--p-trunc-len 150 \

--o-representative-sequences rep-seqs.qza \

--o-table table.qza \

--o-denoising-stats stats.qza

qiime dada2 denoise-paired \

--i-demultiplexed-seqs demux.qza \

--p-trim-left-f 10 \

--p-trim-left-r 10 \

--p-trunc-len-f 150 \

--p-trunc-len-r 150 \

--o-table table.qza \

--o-representative-sequences rep-seqs.qza \

--o-denoising-stats stats.qza

#merging datasets

qiime feature-table merge \

--i-tables table1.qza \

--i-tables table6.qza \

--i-tables table8.qza \

--i-tables table9.qza \

--i-tables table10.qza \

--i-tables table17.qza \

--i-tables table18.qza \

--i-tables table20.qza \

--i-tables table21.qza \

--o-merged-table table.qza

qiime feature-table merge-seqs \

--i-data rep-seqs1.qza \

--i-data rep-seqs6.qza \

--i-data rep-seqs8.qza \

--i-data rep-seqs9.qza \

--i-data rep-seqs10.qza \

--i-data rep-seqs17.qza \

--i-data rep-seqs18.qza \

--i-data rep-seqs20.qza \

--i-data rep-seqs21.qza \

--o-merged-data rep-seqs.qza

#create OTUs

qiime vsearch cluster-features-closed-reference \

--i-table table.qza \

--i-sequences rep-seqs.qza \

--i-reference-sequences 97_otus.qza \

--p-perc-identity 0.97 \

--o-clustered-table table-97.qza \

--o-clustered-sequences rep-seqs-97.qza \

--o-unmatched-sequences unmatched-97.qza

#filter samples

qiime feature-table filter-samples \

--i-table table-97.qza \

--p-min-frequency 1000 \

--p-min-features 4 \

--o-filtered-table table-97-filter1000.qza

qiime feature-table summarize \

--i-table table-97-filter1000.qza \

--o-visualization table-97-filter1000.qzv \

--m-sample-metadata-file Metadata-1-21.txt

#output

qiime tools export \

--input-path table-97-filter1000.qza \

--output-path export-table-97-filter1000

biom convert -i export-table-97-filter1000/feature-table.biom -o export-table-97-filter1000.txt --to-tsv

time qiime feature-classifier classify-sklearn \

--i-classifier 97-gg-classifier.qza \

--i-reads rep-seqs-97.qza \

--o-classification rep-seqs-97-taxonomy.qza

qiime tools export \

--input-path rep-seqs-97-taxonomy.qza \

--output-path rep-seqs-97-taxonomy

qiime taxa barplot \

--i-table table-97-filter1000.qza \

--i-taxonomy rep-seqs-97-taxonomy.qza \

--m-metadata-file Metadata-1-21.txt \

--o-visualization Taxa-bar-plots-97-filter1000.qzv

qiime phylogeny align-to-tree-mafft-fasttree \

--i-sequences rep-seqs-97.qza \

--o-alignment aligned-rep-seqs.qza \

--o-masked-alignment masked-aligned-rep-seqs.qza \

--o-tree unrooted-tree.qza \

--o-rooted-tree rooted-tree.qza

qiime tools export \

--input-path rooted-tree.qza \

--output-path exported-tree

#diversity analysis and visualization

qiime diversity core-metrics-phylogenetic \

--i-phylogeny rooted-tree.qza \

--i-table table-97-filter1000.qza \

--p-sampling-depth 1000 \

--m-metadata-file Metadata-1-21.txt \

--output-dir core-metrics-results

qiime diversity alpha-rarefaction \

--i-table table-97-filter1000.qza \

--i-phylogeny rooted-tree.qza \

--p-min-depth 10 \

--p-max-depth 5000 \

--m-metadata-file Metadata-1-21.txt \

--o-visualization alpha-rarefaction.qzv

#p value-alpha-group-significance

qiime diversity alpha-group-significance \

--i-alpha-diversity core-metrics-results/faith_pd_vector.qza \

--m-metadata-file Metadata-1-21.txt \

--o-visualization core-metrics-results/alpha-faith-pd-group-significance.qzv

qiime diversity alpha-group-significance \

--i-alpha-diversity core-metrics-results/observed_features_vector.qza \

--m-metadata-file Metadata-1-21.txt \

--o-visualization core-metrics-results/alpha-observed_features_vector-group-significance.qzv

qiime diversity alpha-group-significance \

--i-alpha-diversity core-metrics-results/evenness_vector.qza \

--m-metadata-file Metadata-1-21.txt \

--o-visualization core-metrics-results/evenness-group-significance.qzv

qiime diversity alpha-group-significance \

--i-alpha-diversity core-metrics-results/shannon_vector.qza \

--m-metadata-file Metadata-1-21.txt \

--o-visualization core-metrics-results/alpha-shannon_vector-group-significance.qzv

#p value-beta-group-significance

qiime diversity beta-group-significance \

--i-distance-matrix core-metrics-results/bray_curtis_distance_matrix.qza \

--m-metadata-file Metadata-1-21.txt \

--m-metadata-column description \

--o-visualization core-metrics-results/bray_curtis_description-significance.qzv \

--p-pairwise

qiime diversity beta-group-significance \

--i-distance-matrix core-metrics-results/jaccard_distance_matrix.qza \

--m-metadata-file Metadata-1-21.txt \

--m-metadata-column description \

--o-visualization core-metrics-results/jaccard_description-significance.qzv \

--p-pairwise

qiime diversity beta-group-significance \

--i-distance-matrix core-metrics-results/weighted_unifrac_distance_matrix.qza \

--m-metadata-file Metadata-1-21.txt \

--m-metadata-column description \

--o-visualization core-metrics-results/weighted_unifrac_description-significance.qzv \

--p-pairwise

qiime diversity beta-group-significance \

--i-distance-matrix core-metrics-results/unweighted_unifrac_distance_matrix.qza \

--m-metadata-file Metadata-1-21.txt \

--m-metadata-column description \

--o-visualization core-metrics-results/unweighted-unifrac-description-significance.qzv \

--p-pairwise
